# Bedside detection of intracranial midline shift using portable magnetic resonance imaging

**DOI:** 10.1038/s41598-021-03892-7

**Published:** 2022-01-07

**Authors:** Kevin N. Sheth, Matthew M. Yuen, Mercy H. Mazurek, Bradley A. Cahn, Anjali M. Prabhat, Sadegh Salehi, Jill T. Shah, Samantha By, E. Brian Welch, Michal Sofka, Laura I. Sacolick, Jennifer A. Kim, Seyedmehdi Payabvash, Guido J. Falcone, Emily J. Gilmore, David Y. Hwang, Charles Matouk, Barbara Gordon-Kundu, Adrienne Ward RN, Nils Petersen, Joseph Schindler, Kevin T. Gobeske, Lauren H. Sansing, Gordon Sze, Matthew S. Rosen, W. Taylor Kimberly, Prantik Kundu

**Affiliations:** 1 Department of Neurology, Yale School of Medicine, 15 York Street, LLCI Room 1003C, P.O. Box 208018, New Haven, CT 06520 USA; 2 Hyperfine, Inc, Guilford, CT USA; 3 Department of Neuroradiology, Yale School of Medicine, New Haven, CT USA; 4 Department of Neurosurgery, Yale School of Medicine, New Haven, CT USA; 5 Neuroscience Intensive Care Unit, Yale New Haven Hospital, New Haven, CT USA; 6 Athinoula A. Martinos Center for Biomedical Imaging, Massachusetts General Hospital, Charlestown, MA USA; 7 Department of Neurology, Massachusetts General Hospital, Boston, MA USA

**Keywords:** Diagnosis, Medical imaging, Neurology, Stroke

## Abstract

Neuroimaging is crucial for assessing mass effect in brain-injured patients. Transport to an imaging suite, however, is challenging for critically ill patients. We evaluated the use of a low magnetic field, portable MRI (pMRI) for assessing midline shift (MLS). In this observational study, 0.064 T pMRI exams were performed on stroke patients admitted to the neuroscience intensive care unit at Yale New Haven Hospital. Dichotomous (present or absent) and continuous MLS measurements were obtained on pMRI exams and locally available and accessible standard-of-care imaging exams (CT or MRI). We evaluated the agreement between pMRI and standard-of-care measurements. Additionally, we assessed the relationship between pMRI-based MLS and functional outcome (modified Rankin Scale). A total of 102 patients were included in the final study (48 ischemic stroke; 54 intracranial hemorrhage). There was significant concordance between pMRI and standard-of-care measurements (dichotomous, *κ* = 0.87; continuous, *ICC* = 0.94). Low-field pMRI identified MLS with a sensitivity of 0.93 and specificity of 0.96. Moreover, pMRI MLS assessments predicted poor clinical outcome at discharge (dichotomous: adjusted OR 7.98, 95% CI 2.07–40.04*, p* = 0.005; continuous: adjusted OR 1.59, 95% CI 1.11–2.49, *p* = 0.021). Low-field pMRI may serve as a valuable bedside tool for detecting mass effect.

## Introduction

Cerebral edema can develop as a complication of different types of acute brain injuries, including large-territory infarction, spontaneous intracranial hemorrhage, and traumatic brain injury^1–3^. Progressive, space-occupying edema can exert mass effect and displace midline structures^4,5^. Midline shift (MLS) of the brain is a widely recognized marker of mass effect that is associated with poor outcome^5–12^, altered consciousness^11^, and neurological deterioration^5,11,13–15^. The degree of MLS can be used to guide clinical decision-making^5,14^. For example, the presence of MLS greater than 5 mm can serve as a benchmark for emergency surgical evacuation of intracranial hemorrhage^16–18^. Although MLS is often considered the gold standard marker of mass effect^11,12,19^, it is a gross radiologic measure that is less sensitive to smaller morphological changes induced by brain swelling^19^. Other relevant markers of mass effect include the compression of thesal cistern, effacement of the ventricles, and displacement of the brainstem^20^. Additionally, net water uptake^21^ and diffusion-weighted imaging lesion volumes^22^ are important quantitative imaging biomarkers predictive of malignant edema.

Brain swelling and its biomarkers are typically detected and monitored through radiologic imaging studies, such as computed tomography (CT) and magnetic resonance imaging (MRI). However, transport of critically ill patients to designated imaging suites may be challenging or unfeasible, as intrahospital transport is associated with numerous risks and secondary injuries^23–27^. Even in the context of a well-trained transport team, Waydhas reported adverse events occurring 15% of the time patients were transported to a head CT^27^. Consequently, safe and serial access to neuroimaging is limited in large part by the need to transport patients to a remote imaging suite.

Capitalizing on advances in MRI technology, we recently developed and deployed a portable MRI (pMRI) scanner operating at low magnetic field (0.064 T) for the bedside assessment of brain injury in intensive care patients^28^. In another report, we systematically assessed the use of low-field pMRI in obtaining clinically significant imaging of intracerebral hemorrhage^29^. In contrast to high-field MRI systems, low-field pMRI exams can be performed in environments that contain ferromagnetic material, including ventilators, vital signs monitors, and infusion pumps. Despite operation at low-field, this pMRI system can acquire imaging sequences akin to conventional MRI systems, including diffusion-weighted, T2-weighted, T1-weighted, and fluid-attenuated inversion recovery (FLAIR) imaging.

Our recent reports demonstrate the safety and feasibility of pMRI in an intensive care setting^28^ and in the evaluation of intracerebral hemorrhage^29^. However, the utility and applications of pMRI in the assessment of neuropathology remain relatively unexplored. In this study, we evaluated the use of low-field pMRI as a bedside neuroimaging solution for assessing MLS in intensive care patients. We hypothesized that pMRI could be used to identify and quantify MLS as a surrogate of mass effect. Our secondary hypothesis was that pMRI-based midline measurements could predict the neurological outcome of stroke patients.

## Methods

### Study design and participants

In this prospective study, we assessed the ability of pMRI to identify and quantify MLS. This observational study was performed at Yale New Haven Hospital’s Neuroscience Intensive Care Unit (NICU) from July 2018 to July 2020. The pMRI device operated under a research protocol approved by Yale’s Institutional Review Board with an investigational device exemption. All study procedures were performed in accordance with the approved research protocol and relevant guidelines by the Yale Human Research Protection Program.

Patients admitted to the NICU were screened for eligibility. Inclusion criteria entailed a standard-of-care (SOC) non-contrast CT or MRI imaging study indicating ischemic stroke or intracranial hemorrhage (intraparenchymal or subarachnoid hemorrhage). Exclusion criteria included cardiorespiratory instability, patient body habitus exceeding the dimensions of the pMRI scanner (see Technical and Imaging Parameters below), or the presence of at least one of the following MRI contraindications: cardiac pacemakers, insulin pumps, deep brain stimulators, vagus nerve stimulators, and cochlear implants. Eligible patients or their legally authorized representatives were approached for signed informed consent. Clinical data were collected from each participant’s electronic medical record.

### Technical and imaging parameters

**Figure 1:**
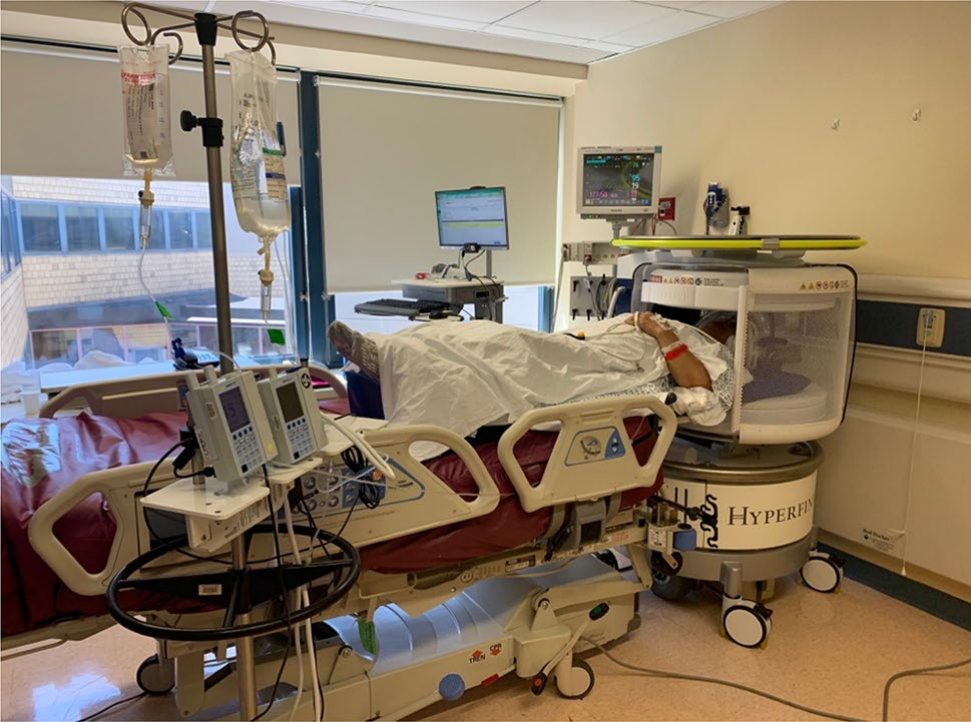
0.064 T Portable MRI scanner in an intensive care unit room. Low-field portable MRI exams were performed in the presence of operational intensive care equipment. The portable MRI operator and bedside nurse were able to remain in the room during scanning. All portable MRI images were available for real-time viewing on an iPad as each sequence was acquired and processed.

We used a 0.064 T MRI system (Hyperfine, Guilford, CT, USA) to obtain pMRI exams at the patient’s bedside (Fig. 1). The pMRI device has a height of 140 cm and a width of 86 cm. The device contains an 8-channel head coil, which has a height of 26 cm and width of 20 cm. The vertical and horizontal clearance of the pMRI are 32 cm and 55 cm, respectively. The scanner uses a biplanar 3-axis gradient system with a peak amplitude of 26 mT/m (on Z-axis) and 25 mT/m (on X- and Y-axis), operates from a standard 110 V, 15A electrical outlet, and does not require any cryogens. All pMRI exams were conducted in single-patient ICU rooms, which included the presence of nearby ferromagnetic equipment (e.g., vital signs monitors, intravenous infusion pumps, ventilators, compressed gas cylinders, and dialysis machines).

The pMRI device is capable of obtaining T2-weighted, T1-weighted, FLAIR, and diffusion-weighted imaging pulse sequences. Image sequences were selected through an electronic interface (iPad Pro third generation). Each acquired sequence was displayed on the iPad in real-time throughout image acquisition and processing. All pMRI images were automatically uploaded in DICOM format to a cloud-based server upon completion of the pMRI exam.

The pMRI system underwent multiple hardware and software updates (hardware Mk1.2, Mk1.5, Mk1.6; software RC3, RC4, RC5, RC6, RC7 RC8) throughout the study. Out of the aforementioned pMRI imaging sequences, T2-weighted and FLAIR images were the most consistent across these updates. Consequently, only T2-weighted (or FLAIR, if a T2-weighted image was not obtained) were analyzed in this study. Whole-brain T2-weighted and FLAIR fast spin echo sequences were acquired in the axial plane. Relevant acquisition parameters were organized as follows (RC3/RC5/RC8): T2-weighted, acquisition time = 8:39/5:28/7:01 min, *TR* = 2000/2000/2200 ms, *TE* = 201/252/253 ms, 1.7 × 1.7 × 5 mm^3^/1.5 × 1.5 × 5 mm^3^/1.5 × 1.5 × 1.5 mm^3^ resolution; FLAIR, acquisition time = 8:35/8:11/9:29 min, *TR* = 1000/100/4000 ms, *TE* = 155/173/228 ms, 1.7 × 1.7 × 5 mm^3^/1.5 × 1.5 × 5 mm^3^/1.6 × 1.6 × 5 mm^3^; 36 slices; field of view 22 cm (anterior/poster) × 18 cm (right/left) × 18 cm (foot/head); number of averages = 1.

Each pMRI scanner must meet factory imaging performance criteria prior to its delivery to a clinical site. These criteria entail the scanning of an image quality phantom to ensure the scanner fulfills performance metrics established by the National Electrical Manufacturers Association (NEMA). System quality assurance results, including metrics on geometric distortion (see Supplementary Material online), are reported to the U.S. Food and Drug Administration. Additionally, each pMRI scanner arrives with an image quality phantom. The phantom is scanned each month on-site, and the phantom images are uploaded to the Hyperfine Cloud Picture Archive and Communication System, which allows for monitoring and evaluation of these phantom images for calibration and quality assurance purposes.

### Imaging analysis

MLS was measured on pMRI exams and, if available, the closest SOC imaging exam (CT or MRI) within 24 h. Three members of the research staff (A.M.P, M.M.Y, M.H.M) with experience in pMRI operation and image analysis used Horos (v.3.3.5) to perform MLS measurements. Raters first annotated pMRI images before annotating SOC images. Raters were blinded to clinical data and patient identifiers.

**Figure 2:**
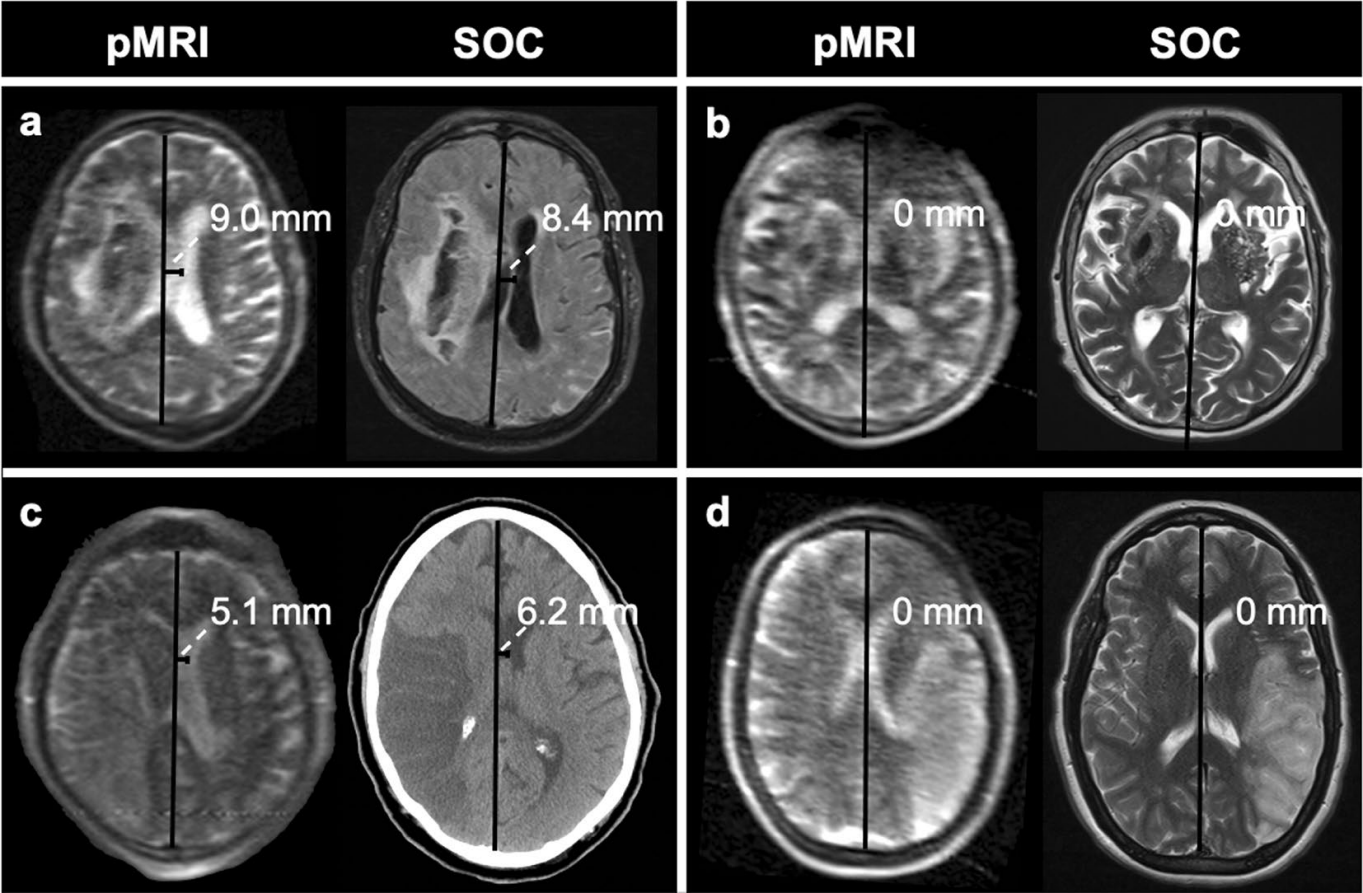
Example midline shift measurements on portable MRI (pMRI) and standard-of-care (SOC) imaging exams. (**a**) 81-year-old male with right intracerebral hemorrhage. Midline shift was measured to be 9.0 mm and 8.4 mm on the pMRI T2-weighted (T2W) and standard-of-care (SOC) MRI fluid-attenuated inversion recovery (FLAIR) images, respectively. (**b**) 43-year-old male with right intracerebral hemorrhage. No midline shift was measured on either pMRI T2W or SOC MRI T2W exams. (**c**) 71-year-old male with right M1 occlusion. Midline shift was measured to be 5.1 mm and 6.2 mm on the pMRI T2W and SOC CT images, respectively. (**d**) 44-year-old female with left M2 occlusion. No midline shift was measured on either pMRI T2W or SOC MRI T2W exams. Figure created using: Microsoft PowerPoint, Version 16.52, https://www.microsoft.com/en-us/microsoft-365/powerpoint.

Following previously published approaches in stroke populations^10,30,31^, MLS was defined as any deviation of the septum pellucidum from the midline. MLS was assessed as a continuous and dichotomous (present or absent) variable. Continuous MLS measurements were obtained by drawing a line from the anterior and posterior attachments of the falx cerebri and then drawing a second, perpendicular line to the septum pellucidum at the point of maximal deviation. MLS was measured as the length in millimeters of the second line (continuous variable) (Fig. 2)^11,30–33^. MLS greater than 2 mm is associated with poor clinical outcome^34,35^, so we defined any rater’s MLS measurement ≥ 2 mm to indicate the presence of significant MLS (dichotomous variable). For both pMRI and SOC MLS measurements, we generated a consensus (dichotomous) and averaged (continuous) MLS assessment for each patient. The consensus (present or absent) MLS assessment was obtained from the majority consensus of the three raters’ dichotomous MLS assessments. The averaged MLS assessment was obtained by averaging the three raters’ continuous MLS measurements.

### Neurological outcome

Functional outcome at discharge was assessed by the modified Rankin Scale (mRS). The mRS scale ranges from 0 (no residual stroke symptoms) to 6 (death), and mRS scores were dichotomized into good (0–3) and poor clinical outcomes (4–6)^36^.

### Statistics

We present categorical variables as numbers (%) and continuous variables as mean (standard deviation [SD]) or median (interquartile range [IQR]), as appropriate. Interrater reliability between the raters’ pMRI and SOC MLS assessments was computed using the Fleiss kappa (*κ*) statistic for dichotomous MLS assessments and the intraclass correlation coefficient (*ICC*) for continuous MLS measurements.

Low-field pMRI-based MLS measurements were compared to SOC-based measurements, which were considered the ground truth. We first compared each raters’ individual measurements (*κ* for dichotomous, *ICC* for continuous). We then compared majority consensus and averaged MLS measurements between pMRI and SOC imaging studies (*κ* for dichotomous, *ICC* for continuous). The agreement between pMRI and SOC MLS assessments was also studied by the Bland–Altman method with calculation of bias and limits of agreement. To account for confounding effects due to continuous development of the pMRI system, identical analyses were performed in three groups of patients: patients scanned using software versions RC3 and RC4, patients scanned using software versions RC5 and RC6, and patients scanned using software versions RC7 and RC8.

To assess the relationship between MLS and discharge functional outcome, we performed a *χ*^2^ test to see if there was an association between the presence of MLS and discharge mRS scores. We then assessed the relationship between dichotomous and continuous MLS assessments and clinical outcomes using unadjusted and adjusted binary logistic regression models. We expressed the effect of MLS on functional outcome as unadjusted and adjusted common odds ratios (cOR and acOR, respectively). In stratified analyses, we evaluated ischemic stroke (IS) and intracranial hemorrhage (ICH) patients separately. To adjust forseline prognostic variables, our adjusted models included sex, race, age, stroke severity (NIH Stroke Scale score at admission), history of diabetes mellitus, atrial fibrillation, and prior stroke. All statistical analyses were performed using RStudio version 1.2.5033.

## Results

### Patient characteristics

We obtained pMRI exams on 105 patients (median [IQR] age, 64 [53–74] years; 51 female [49%]; range body mass index, 16–46) with ischemic stroke (IS) and intracranial hemorrhage (ICH). No adverse events or complications occurred. Out of the 105 patients examined, three patients were excluded from further analysis. Two of these patients were excluded due to motion degradation of their pMRI exams. One patient had their pMRI exam terminated early due to claustrophobia, precluding either a T2-weighted or FLAIR image from being obtained. Of the 102 patients included in the final cohort (Table 1), 48 (47%) patients presented with IS, and 54 (53%) patients presented with ICH (36 intraparenchymal hemorrhages, 18 subarachnoid hemorrhages).

**Table 1:** Patient demographics and clinical characteristics.

Characteristics	All patients	Ischemic stroke	Intracranial hemorrhage
Total no	102	48	54
Age, median (IQR), y	64 (53–74)	64 (56–76)	64 (51–73)
Female, no. (%)	50 (49)	21 (44)	29 (54)
Race, no. (%)^a^			
White	73 (72)	39 (81)	34 (63)
Black/African American	16 (16)	6 (13)	10 (19)
Asian	7 (7)	2 (4)	5 (9)
Pacific Islander	1 (1)	0 (0)	1 (2)
Other	5 (5)	1 (2)	4 (7)
Baseline medical history, no. (%)^b,c^			
Atrial fibrillation	13 (13)	8 (17)	5 (9)
Diabetes mellitus	16 (16)	10 (21)	6 (11)
Hypertension	56 (55)	30 (63)	26 (48)
Hyperlipidemia	34 (33)	20 (42)	14 (26)
Prior stroke	13 (13)	6 (13)	7 (13)
LKN to exam, median (IQR), h	65 (41–120)	54 (34–98)	72 (43–161)
Presence of MLS at exam, no. (%)^d^	22 (22)	11 (23)	11 (20)
Admission NIHSS, median (IQR)	4 (1–12)	8 (2–18)	1 (0–7)
Discharge mRS, median (IQR)	3 (1–4)	4 (1–4)	3 (1–4)

IQR, interquartile range; y, year; hr, hour; LKN, last known normal; NIHSS, NIH Stroke Scale; mRS, modified Rankin Scale; MLS, midline shift.

^a^Percentages may not total to 100% because of rounding.

^b^Seline medical history information was unavailable for one patient.

^c^LKN to exam and admission NIHSS information was unavailable for one patient.

^d^Presence of MLS at exam was determined by assessments made on portable MRI images.

Based on pMRI MLS measurements, 22 (22%) patients had MLS at the time of their pMRI exam (median [IQR] time between last known normal and pMRI exam, 65 [41–120] hours), with an average of 3.63 ± 1.80 [SD] mm. In particular, 11 (23%) of the IS patients exhibited MLS, with an average of 3.08 mm ± 0.65 mm, and 11 (20%) of the ICH patients showed MLS, with an average of 4.17 ± 2.39 mm.

We recorded pMRI examination times for five non-intubated stroke patients; these recorded times are also noted in a different report^29^. Single-sequence (T2-weighted or FLAIR) pMRI exams were obtained in 21:00 ± 0:10 [SD] minutes. Point-of-care pMRI exams required 8:33 ± 0:09 min for set-up, which entailed bringing the scanner into the room, positioning the pMRI behind the patient’s bed, and boosting the patient into the scanner. T2-weighted and FLAIR sequences were acquired in 7:01 ± 0:06 and 8:45 ± 0:03 min, respectively. After the imaging protocol was completed, removing the patient from the scanner and restoring the patient’s room to the prior state required 4:27 ± 0:03 min.

### Interrater reliability and accuracy of midline shift measurements

MLS was measured by three raters on the pMRI exams of 102 patients and the closest SOC imaging exam within 24 h. A total of 66 patients received a SOC imaging exam within 24 h of their pMRI exam (34 MRI and 32 CT). Specifically, 9 patients received a pMRI exam prior to their SOC imaging exam (mean time [SD] between exams, 13 [9] hours); 57 received a pMRI exam after their SOC imaging exam (mean time [SD] between exams, 12 [7] hours). There was significant interrater agreement for pMRI (*κ* = 0.64, *p* = 0.000, *ICC* = 0.93, *p* = 1.7 × 10^–35^) and SOC MLS measurements (*κ* = 0.77, *p* = 0.000, *ICC* = 0.95, *p* = 3.2 × 10^–45^).

**Figure 3:**
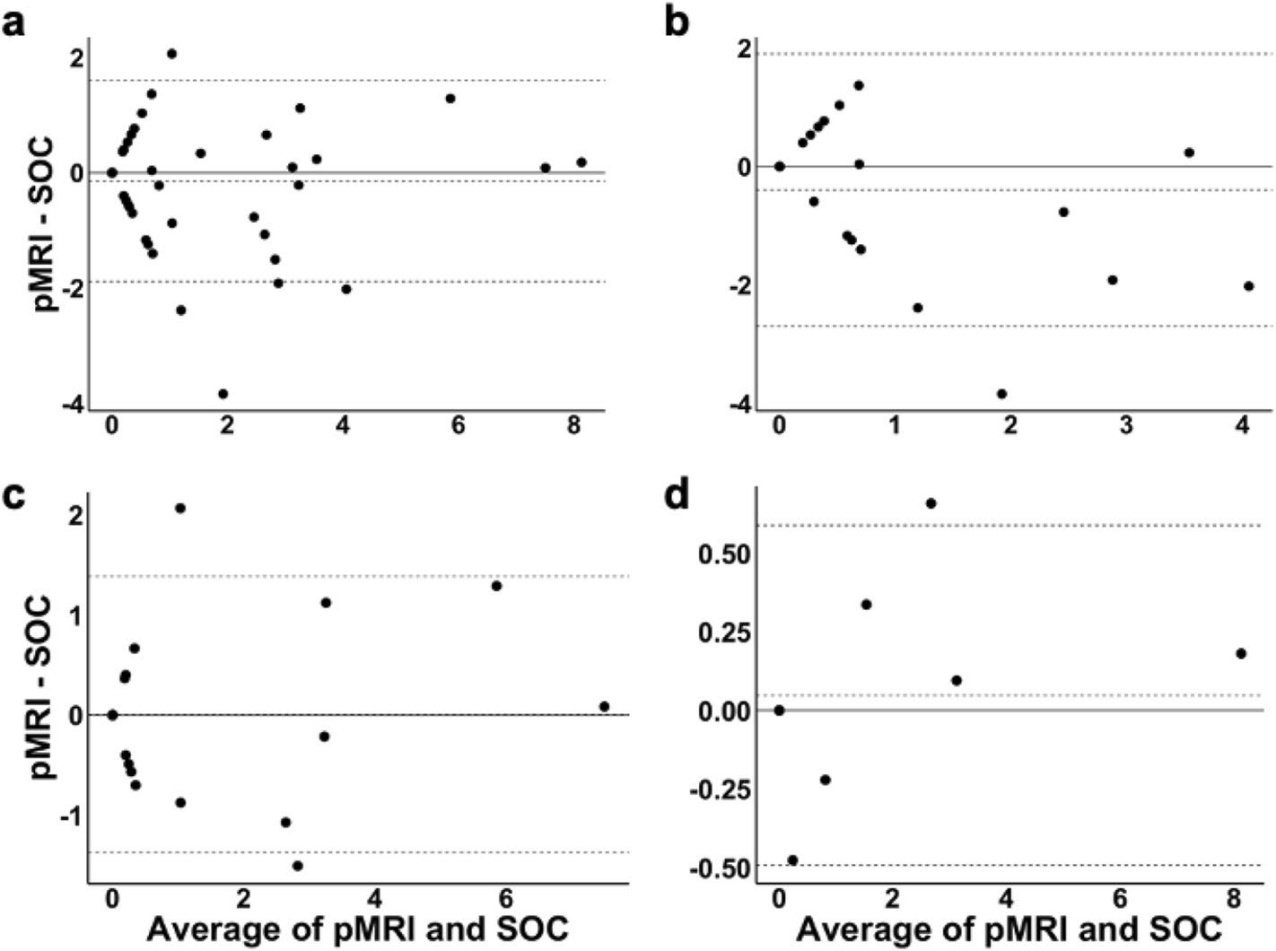
Bland–Altman plots of averaged portable MRI (pMRI) and standard-of-care (SOC) midline shift (MLS) assessments. (**a**) The Bland–Altman plot of pMRI and SOC MLS assessments for all patients demonstrated a bias of − 0.14 mm and limits of agreement from 1.60 mm to − 1.89 mm. Four measures (6%) were outside the limits of agreement. (**b**) The Bland–Altman plot of pMRI and SOC MLS assessments for patients scanned using pMRI software versions RC3 and RC4 showed a bias of − 0.40 mm and limits of agreement from 1.90 mm to − 2.70 mm. One measure (4%) was outside the limits of agreement. (**c**) The Bland–Altman plot of pMRI and SOC MLS assessments for patients scanned using pMRI software versions RC5 and RC6 showed a bias of 0.01 mm and limits of agreement from 1.39 mm to − 1.37 mm. Two measures (7%) were outside the limits of agreement. (**d**) The Bland–Altman plot of pMRI and SOC MLS assessments for patients scanned using pMRI software versions RC7 and RC8 demonstrated a bias of 0.05 mm and limits of agreement from 0.59 mm to − 0.49 mm. One measure (8%) was outside the limits of agreement. Figure created using: (1) RStudio Team (2019). RStudio: Integrated Development for R. RStudio, Inc., Boston, MA URL http://www.rstudio.com/, (2) Microsoft PowerPoint, Version 16.52, https://www.microsoft.com/en-us/microsoft-365/powerpoint.

For the 66 patients that received a SOC imaging exam within 24 h of their pMRI exam, we assessed the agreement between pMRI-based and SOC-based MLS assessments. For each rater, there was significant agreement between pMRI-measured and SOC-measured MLS assessments: Rater 1 (*κ* = 0.74*, p* = 4.0 × 10^–10^; *ICC* = 0.90, *p* = 1.0 × 10^–11^), Rater 2 (*κ* = 0.67, *p* = 2.9 × 10^–8^; *ICC* = 0.90, *p* = 1.0 × 10^–15^), Rater 3 (*κ* = 0.57*, p* = 3.5 × 10^–6^; *ICC* = 0.85*, p* = 7.7 × 10^–13^)**.** For consensus (dichotomous) and averaged (continuous) measurements, we found significant agreement between pMRI-measured and SOC-measured MLS assessments (*κ* = 0.87*, p* = 1.7 × 10^–12^; *ICC* = 0.94*, p* = 5.9 × 10^–23^) (Table 2). The Bland–Altman plot of averaged pMRI and SOC MLS assessments showed a bias of − 0.14 mm and limits of agreement from 1.60 mm to − 1.89 mm (Fig. 3a). With SOC-measured MLS assessments as the ground-truth, low-field pMRI detected the presence of significant MLS with a sensitivity of 0.93 and specificity of 0.96.

**Table 2:** Detection and measurement of midline shift using portable MRI.

	Sensitivity	Specificity	Dichotomous pMRI vs. SOC (*κ*)	Continuous pMRI vs. SOC (*ICC)*
All SW Versions	0.93	0.96	0.87	0.94
RC3/RC4^a^	0.80	0.95	0.75	0.73
RC5/RC6^b^	1.00	0.96	0.90	0.97
RC7/RC8^c^	1.00	1.00	1.00	1.00

SW, software; *κ*, kappa statistic; *ICC*, intraclass correlation coefficient; pMRI, portable MRI; SOC, standard-of-care imaging.

^a^Patients scanned using pMRI software versions RC3 and RC4.

^b^Patients scanned using pMRI software versions RC5 and RC6.

^c^Patients scanned using pMRI software versions RC7 and RC8.

To account for confounding effects due to evolving improvements of the pMRI system, the abovementioned analyses were performed in three groups of patients: patients scanned using pMRI software versions RC3 and RC4, patients scanned using pMRI software versions RC5 and RC6, and patients scanned using pMRI software versions RC7 and RC8.

For patients scanned using software versions RC3 and RC4 (n = 26), the agreement between pMRI-measured and SOC-measured MLS assessments was *k* = 0.75, *p* = 1.3 × 10^–4^ and *ICC* = 0.73, *p* = 6.3 × 10^–4^ (Table 2). The Bland–Altman plot of averaged pMRI and SOC MLS assessments showed a bias of − 0.40 mm and limits of agreement from 1.90 mm to − 2.70 mm (Fig. 3b). For this group of patients, low-field pMRI detected the presence of significant MLS with a sensitivity of 0.80 and specificity of 0.95.

For patients scanned using software versions RC5 and RC6 (n = 28), the agreement between pMRI-measured and SOC-measured MLS assessments was *k* = 0.90, *p* = 1.7 × 10^–6^ and *ICC* = 0.97, *p* = 6.4 × 10^–14^ (Table 2). The Bland–Altman plot of averaged pMRI and SOC MLS assessments showed a bias of 0.01 mm and limits of agreement from 1.39 mm to − 1.37 mm (Fig. 3c). For this group of patients, low-field pMRI detected the presence of significant MLS with a sensitivity of 1.00 and specificity of 0.96.

For patients scanned using software versions RC7 and RC8 (n = 12), the agreement between pMRI-measured and SOC-measured MLS assessments was *k* = 1.00, *p* = p = 5.3 × 10^–4^ and *ICC* = 1.00, *p* = 1.6 × 10^–12^ (Table 2). The Bland–Altman plot of averaged pMRI and SOC MLS assessments demonstrated a bias of 0.05 mm and limits of agreement from 0.59 mm to − 0.49 mm (Fig. 3d). For this group of patients, low-field pMRI detected the presence of significant MLS with a sensitivity of 1.00 and specificity of 1.00.

**Figure 4:**
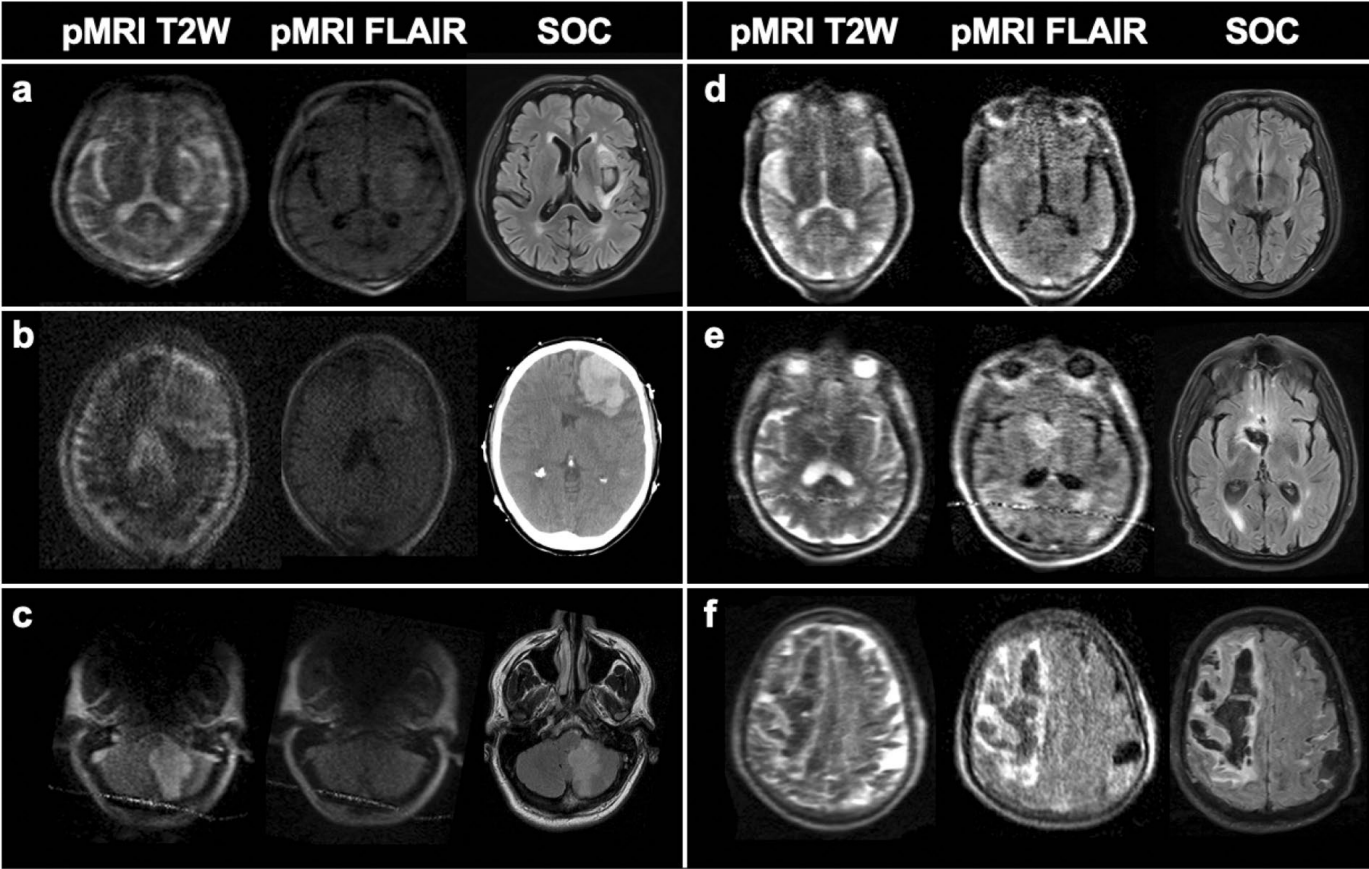
Evolving image quality and continuous development of the portable MRI (pMRI) device. (**a**) 71-year-old male with leftsal ganglia intracerebral hemorrhage; software RC3. (**b**) 71-year-old female with left frontal intracerebral hemorrhage; software RC4. (**c**) 58-year-old male with left cerebellar infarct; software RC5. The pMRI fluid-attenuated inversion recovery (FLAIR) did not capture the lesion. (**d**) 50-year-old male with right middle cerebral artery infarct; software RC6. (**e**) 68-year-old male with rightsal ganglia intracerebral hemorrhage; software RC7. (**f**) 81-year-old male with right frontoparietal intracerebral hemorrhage; software RC8. All SOC MRI exams shown are FLAIR images. T2W indicates T2-weighted; SOC indicates standard-of-care imaging. Figure created using: Microsoft PowerPoint, Version 16.52, https://www.microsoft.com/en-us/microsoft-365/powerpoint.

There was substantial agreement of pMRI-based and SOC-based MLS assessments for all software versions, but continuous improvement in pMRI image quality (Fig. 4) corresponded with increased diagnostic capability of the pMRI to accurately detect and measure midline shift.

### Midline shift and functional outcome

**Figure 5:**
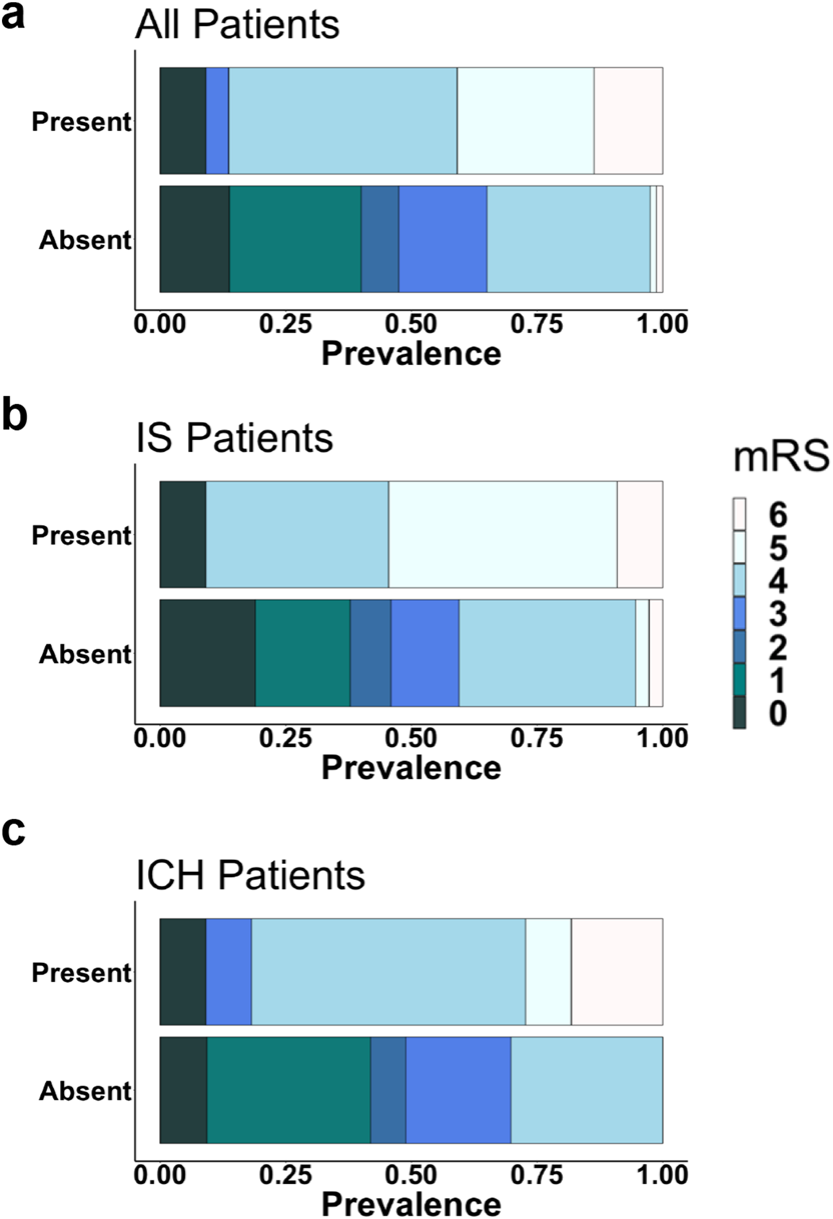
Modified Rankin Scale distributions for patients presenting with and without midline shift. Presence of midline shit was significantly associated with discharge functional outcome (mRS) for (**a**) all patients (*χ*^2^ = 34.29, *p* = 0.000), (**b**) ischemic stroke (IS) patients only (*χ*^2^ = 18.18, *p* = 0.006), and (**c**) intracranial hemorrhage (ICH) patients only (*χ*^2^ = 18.21, *p* = 0.006). Figure created using: (1) RStudio Team (2019). RStudio: Integrated Development for R. RStudio, Inc., Boston, MA URL http://www.rstudio.com/, (2) Microsoft PowerPoint, Version 16.52, https://www.microsoft.com/en-us/microsoft-365/powerpoint.

We found a significant association between dichotomous pMRI-based MLS assessments (present or absent) and discharge functional outcome (mRS). This association was seen for all patients (*χ*^2^ = 34.29, *p* = 5.9 × 10^–6^), IS patients only (*χ*^2^ = 18.18, *p* = 0.006), and ICH patients only (*χ*^2^ = 18.21 *p* = 0.006) (Fig. 5). Presence of significant MLS predicted poor discharge functional outcome in both unadjusted and adjusted binary logistic regressions. This effect of MLS presence on outcome was seen for all patients (cOR, 11.76, *p* = 2.1 × 10^–4^; acOR, 7.98, *p* = 0.005), IS patients only (cOR, 14.67, *p* = 0.015; acOR, 50.47, *p* = 0.027), and ICH patients only (cOR, 10.04, *p* = 0.006; acOR, 16.37, *p* = 0.033) (Table 3).

**Table 3:** Midline shift on portable MRI predicts poor discharge functional outcome.

MLS assessment^a^	Patient cohort	cOR (95% CI)	*P* value	acOR^b^ (95% CI)	*P* value
Dichotomous	All Patients	11.76 (3.62–53.20)	2.1 × 10^–4^	7.98 (2.07–40.04)	0.005
	IS	14.67 (2.43–> 100)	0.015	50.47 (2.91–> 100)	0.027
	ICH	10.04 (2.29–74.69)	0.006	16.37 (1.56–> 100)	0.033
Continuous	All Patients	1.80 (1.30–2.70)	0.002	1.59 (1.11–2.49)	0.021
	IS	1.96 (1.17–3.88)	0.022	2.22 (0.96–5.13)	0.063
	ICH	1.72 (1.17–3.01)	0.021	1.74 (1.05–3.3.0)	0.048

cOR, common odds ratio; acOR, adjusted common odds ratio; IS, ischemic stroke; ICH, intracranial hemorrhage; MLS, midline shift.

^a^Portable MRI-based MLS assessments.

^b^Multivariable binary logistic regression model, adjusting for patient age, sex, race, stroke severity (NIH Stroke Scale score at admission), history of diabetes mellitus, atrial fibrillation, and prior stroke.

We assessed the relationship between continuous MLS measurements and discharge functional outcome. Continuous MLS assessments predicted poor discharge functional outcome in both unadjusted and adjusted binary logistic regressions (cOR, 1.80, *p* = 0.002; acOR, 1.59, *p* = 0.021). For IS patients, continuous assessments predicted poor discharge functional outcome in our unadjusted model (cOR, 1.96, *p* = 0.022) and trended towards significance in our adjusted model (acOR, 2.22, *p* = 0.063). For ICH patients, continuous MLS assessments predicted poor discharge functional outcome (cOR, 1.72, *p* = 0.021; acOR, 1.74, *p* = 0.048) (Table 3).

## Discussion

We report the use of low-field pMRI for bedside assessment of MLS in patients with IS and ICH. This approach enabled the acquisition of bedside neuroimaging exams that visualized MLS, a well-known marker of mass effect and cerebral edema^5,10,11,37^. We show that MLS measurements on pMRI images are consistent with measurements obtained on conventional MRI and CT studies. We also demonstrate that MLS on pMRI neuroimaging is associated with worse discharge functional outcome, recapitulating a well-established clinical relationship^5–11^.

Neuroimaging studies are integral to the initial assessment and neurological monitoring of patients with acute brain injuries. In conventional imaging pathways, patients must be transported to a dedicated imaging suite. However, intrahospital transport of patients is associated with numerous cardiovascular and respiratory risks^23–27^, which may render the acquisition of conventional CT or MRI imaging unfeasible for clinically unstable patients^38^. MRI scanners operating at low-field magnetic strength enable scanning outside of traditional imaging suites, as they are compatible with nearby ferromagnetic material. While previous approaches in low-field MRI, such as pre-polarized MRI, have been explored^39^, there has not been a low-field MRI device for head imaging that is entirely portable and has been successfully deployed in a clinical environment.

We previously reported the first use of a highly mobile low-field pMRI device to obtain head imaging at the bedside of intensive care patients^28^ and to evaluate intracerebral hemorrhage^29^. The current study extends our understanding of the unique applications of pMRI in evaluating neuropathology at the bedside. In brain-injured patients, attribution of a change in the level of arousal often requires neuroimaging to diagnose MLS, a well-known marker of mass effect and brain-swelling^5,10,11,37^. MLS is one of multiple important biomarkers for acute brain injuries, and the detection of significant MLS can serve as a radiologic indicator for treatment with hyperosmolar agents or neurosurgical interventions, such as decompressive craniectomy and hematoma evacuation. Monitoring changes in MLS is also important when evaluating the efficacy of such treatments, as unresolved MLS predicts worse clinical outcome^40–42^ while reversal of MLS is associated with improved consciousness and survival^43–45^. Our data show that pMRI can identify and quantify MLS with clinically significant accuracy, demonstrating the unique utility of low-field pMRI as a bedside tool for monitoring MLS. Further study is required to assess the ability of pMRI to detect smaller morphological markers of mass effect, includingsal cistern compression, ventricle effacement, and brainstem displacement.

Our study has several limitations. First, pMRI and conventional imaging studies were not obtained simultaneously, with an average time difference of 13 ± 8 [SD] hours. Since MLS is a dynamic neurological marker, this limitation may have induced discrepancies between the pMRI and conventional MLS assessments. Second, it is important to note that patients under 18 years of age and those with a cardiovascular implantable device were not included in this study, so these results cannot be extrapolated to those populations. However, given recent reports of safe, feasible MRI at 1.5 T for patients with cardiovascular implants^46–49^, using a low magnetic field pMRI in this patient population is theoretically possible and requires further study. Finally, patients were imaged at a single-center ICU. Replication of these findings at multiple centers and in clinical environments outside an intensive care setting (e.g., emergency medicine) is necessary before generalizing the results of the current study.

Our approach has several unique aspects. First, we successfully deployed an innovative MRI technology that enabled neuroimaging at the bedside. Many brain pathologies, like MLS, evolve over a dynamic time window and, in turn, require serial imaging. Similarly, neurosurgical interventions often require preoperative and postoperative imaging. Repeated transport of critically ill patients to neuroimaging suites may be unfeasible and hazardous. In a reversal of the current imaging paradigm, we deployed a pMRI directly to the bedside of stroke patients and acquired whole-brain imaging that detected MLS within 10 min (7:01 min for T2W, 8:45 min for FLAIR). Point-of-care pMRI can serve as a safe and viable approach to neuroimaging when serial transport to conventional imaging suites is otherwise contraindicated.

Additionally, pMRI operates on a low-field magnetic strength. Traditional MRI systems operate on high-field magnetic strength, requiring rigid safety precautions each time a healthcare worker enters an MRI suite. These constraints, in addition to the enclosed design of traditional MRI scanners, prevent healthcare workers from easily accessing and caring for patients during conventional MRI exams. In our study, the low-field magnetic strength of the pMRI allowed nurses to freely enter and exit the patient’s ICU room during bedside exams without projectile risk. Moreover, the open geometry design of the pMRI enabled nurses to directly contact and care for the patient (e.g., temperature monitoring, intravenous injection of medication) throughout the imaging exam. These results outline the potential use of pMRI for intensive care patients that require frequent attention and care.

It is important to contextualize the use of low-field pMRI with other portable imaging techniques, including portable CT (pCT) and transcranial ultrasound imaging. Both pCT and ultrasound imaging have been used to detect MLS of the brain at the bedside^50,51^. Ultrasound imaging is an accessible bedside technique that is best suited for monitoring cerebral blood flow and vessel imaging. However, ultrasound’s capacity for structural imaging is limited by the distortion of ultrasound beams as they cross the skull^52^. Point-of-care pCT is a well-explored imaging modality that can provide valuable imaging at the bedside^53^. Point-of-care pCT scanners are capable of non-contrast CT, CT angiography, and CT perfusion, which enables the detection of both anatomical lesions, including hemorrhage^54^ and subacute ischemia^55^, and vascular abnormalities, such as an ischemic penumbra^56^ and large-vessel occlusions^57^. Similar to pCT scanners, the low-field pMRI evaluated in this report can detect hemorrhage and ischemia. The low-field pMRI device can also detect restricted diffusion through diffusion-weighted imaging^28^. However, the low-field pMRI device does not currently have MR angiography or perfusion weighted imaging, limiting its ability to detect ischemic penumbras and large-vessel occlusions.

Low-field pMRI and pCT are the two most analogous portable imaging techniques, but there are several limitations to bedside pCT which have prevented its widespread adoption in dynamic hospital settings. Compared to fixed CT scanners, pCT devices have lower spatial resolution, amplified noise, and higher radiation risks^58–60^. Moreover, bedside pCT requires highly trained technicians and lead shielding around the point-of-care, limiting its ease of use. In contrast, low-field pMRI does not use any ionizing radiation nor require specialized MRI technicians for use. In this study, all pMRI exams were conducted by research assistants under the supervision of nearby nurses. Moreover, pMRI exams were configured by simply connecting an iPad to the pMRI’s local hotspot and selecting a pre-configured imaging protocol on a user interface hosted on a web browser, demonstrating pMRI’s ease of use. Finally, pCT captures only one type of structural image, while pMRI can obtain multiple imaging sequences, including T2-weighted, FLAIR, and diffusion-weighted imaging^28^. Compared to pCT, the unique strengths of pMRI lie within its capacity to obtain multimodal imaging in a safe and feasible manner.

Our study demonstrates the use of a highly mobile low-field MRI scanner to detect clinically significant MLS at the patient’s bedside. Future studies will need to delineate the strengths and limitations of pMRI scanning for different timepoints of brain injury, patient populations, and medical environments. Further validation of the pMRI’s sensitivity and specificity to other intracranial pathologies, such as ischemic stroke and intracranial hemorrhage, is also required. Nonetheless, the current results demonstrate the clinical feasibility of pMRI in a complex medical environment^28^, and we report the first assessment of MLS as a surrogate of mass effect using a portable, bedside MRI device. In instances where single or repeated transport of patients to conventional imaging studies is unfeasible, point-of-care pMRI may serve as a valuable bedside tool that can facilitate the study of disease processes over a dynamic profile.

## Supplementary Information

Supplementary Information.

## Data Availability

Anonymized data is available to qualified researchers upon reasonable request.
