# Supplementary material for: Bedside detection of intracranial midline shift using portable magnetic resonance imaging
Source: Sci Rep. 2022 Jan 7;12:67. doi: 10.1038/s41598-021-03892-7 (PMC8742125; doi:10.1038/s41598-021-03892-7)
Supplement: Supplementary file 1 — Supplementary Information. [file 41598_2021_3892_MOESM1_ESM.docx]

**Bedside Detection of Intracranial Midline Shift Using**

**Portable Magnetic Resonance Imaging**

Kevin N. Sheth, MD^1*^, Matthew M. Yuen BA^1^, Mercy H. Mazurek BS^1^, Bradley A. Cahn BS^1^, Anjali M. Prabhat BA^1^, Sadegh Salehi PhD^2^, Jill T. Shah BA^1^, Samantha By PhD^2^, E. Brian Welch PhD^2^, Michal Sofka PhD^2^, Laura I. Sacolick PhD^2^, Jennifer A. Kim MD, PhD^1^, Seyedmehdi Payabvash MD^3^, Guido J. Falcone MD^1^, Emily J. Gilmore MD^1^, David Y. Hwang MD^1^, Charles Matouk MD^4^, Barbara Gordon-Kundu MD^1^, Adrienne Ward RN^5^, Nils Petersen MD, PhD^1^, Joseph Schindler MD^1^, Kevin T. Gobeske MD, PhD^1,^ Lauren H. Sansing MD^1^, Gordon Sze MD^3^, Matthew S. Rosen PhD^6^, W. Taylor Kimberly MD PhD^7^, Prantik Kundu PhD^2^

Affiliations:

^1^Department of Neurology, Yale School of Medicine, New Haven, CT, USA

^2^Hyperfine, Inc, Guilford, CT, USA

^3^Department of Neuroradiology, Yale School of Medicine, New Haven, CT, USA

^4^Department of Neurosurgery, Yale School of Medicine, New Haven, CT, USA

^5^Neuroscience Intensive Care Unit, Yale New Haven Hospital, New Haven, CT, USA

^6^Athinoula A. Martinos Center for Biomedical Imaging, Massachusetts General Hospital,

Charlestown, MA, USA

^7^Department of Neurology, Massachusetts General Hospital, Boston, MA, USA

***Correspondence:**

Kevin N. Sheth, MD

15 York Street, LLCI Room 1003C

P.O. Box 208018

New Haven, CT 06520, USA

Phone: 203-737-8051

Email: [kevin.sheth@yale.edu](mailto:kevin.sheth@yale.edu) |

**SUPPLEMENTARY METHODS**

Geometric distortion was quantified and mapped in accordance with the National Electrical Manufacturers Association (NEMA) standard.^1^ System quality assurance phantoms were scanned by three portable MRI scanners, three runs each. The phantom is custom designed for assessing image quality. The phantom fits into the Hyperfine scanner Head Coil and approximates the dimensions of an adult human head. The phantom enclosure and features are acrylic plastic, and the phantom is filled with a solution of distilled water and 0.5 g/L copper sulfate. The inner filled dimensions are 200x160x180 mm^3^. Inside the phantom are structures designed to facilitate a variety of tests of scanner performance. The imaging sequence used for acquisition is a 3D inversionless fast spin echo with *TR* = 1000 ms, *TE* = 6 ms, and 48 echoes (**Supplementary Fig. 1a**). The sequence has a field of view of 24 cm (anterior/posterior) x 22 cm (right/left) and 22 cm (foot/head) and resolution was 2x2x2 mm^3^.

Prior to measuring geometric distortion, a rigid translation and rotation was computed and applied to remove positioning errors from the measurement. Geometric error was then computed for a spherical volume of 16 cm diameter (**Supplementary Fig. 1b**) using the Python SimpleITK Registration toolset (<http://www.simpleitk.org>) (parameters outlined in **Supplementary Table 1**). Specifically, geometric distortion was measured from the computed displacement field that relates the acquired three-dimensional image to a three-dimensional model image of the phantom (**Supplementary Fig. 1c)**. The model was verified to accurately represent the phantom by an external 3D Computed Tomography measurement of the phantom with accuracy of ≤ 1 mm. Error was computed for each voxel in the acquired image. Average distortion is reported for concentric spheres of 10 mm from the magnet isocenter outwards (**Supplementary Table 1**), as described previously.^1^ The radial distance between the magnet isocenter and point of MLS measurement (maximal deviation of the septum pellucidum) was measured using Horos (v.3.3.5) for all patients imaged with software versions RC6 onwards (n = 31) (**Supplementary Fig. 1d).**

**Supplementary Reference:**

1 National Electrical Manufacturers Association Standards Publication MS 12-2016, Quantification and Mapping of Geometric Distortion for Special Applications. Published March 2017. Accessed 10 September 2021. https://www.nema.org/standards/view/quantification-and-mapping-of-geometric-distortion-for-special-applications

**
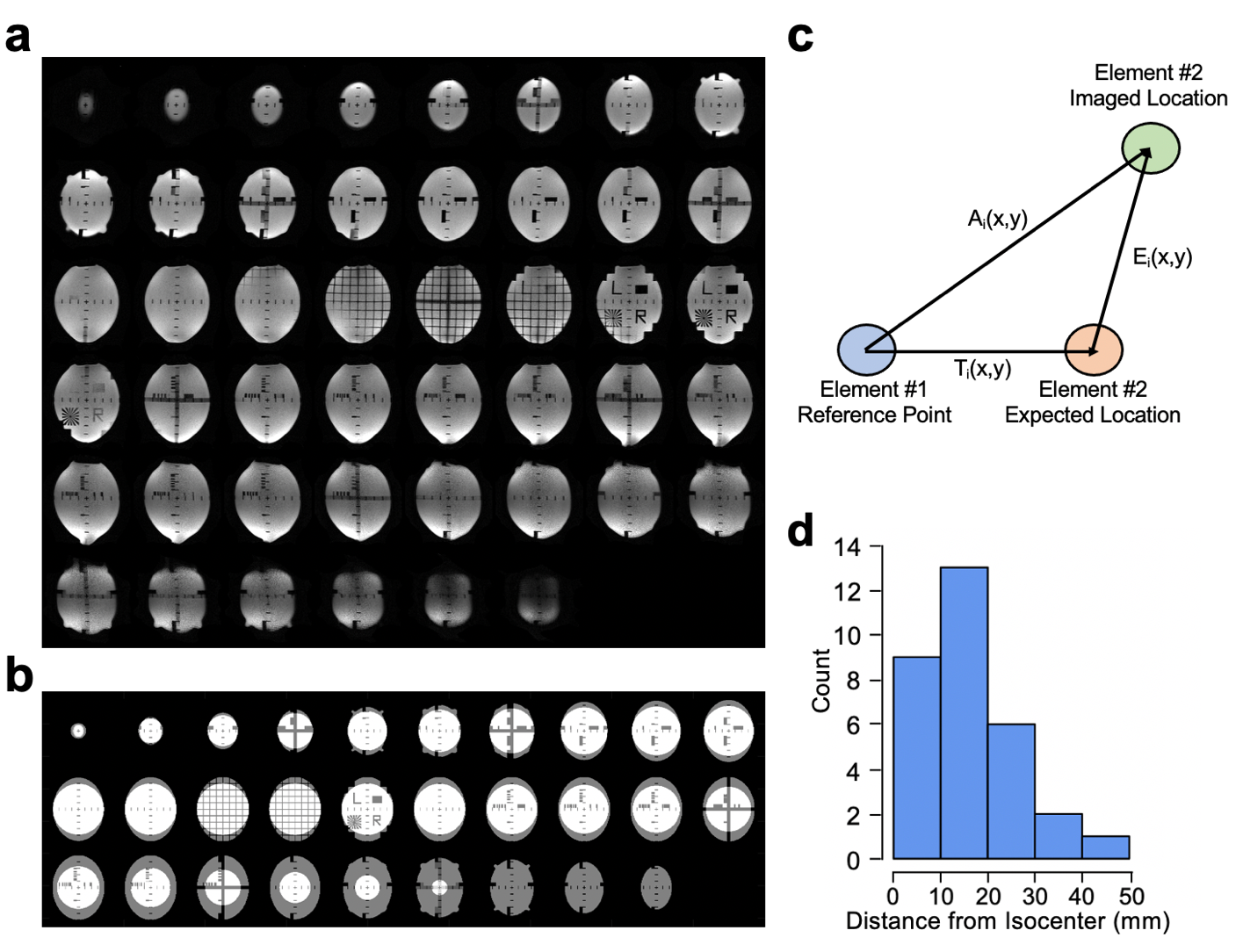
Supplementary Figure 1**

Geometric distortion on portable MRI. **(a)** Example image of phantom. Every other slice is shown. **(b)** Multi-slice plots of the phantom model image. The geometric distortion measurement region is indicated by the white overlay (16 cm diameter sphere). The phantom’s center is located in the center of the slice showing the ‘L’ and ‘R’ letters. **(c)** Geometric error definition reproduced from the NEMA Standards Publication MS 12-2010.^1^ Two elements with an Apparent Spacing of A_i_(x,y) but a True Spacing of T_i_(x,y). E_i_(x,y) is the Error between the True and Acquired position. The chosen reference point is the portable MRI’s magnet isocenter. **(d)** The radial distance of MLS measurements from the magnet isocenter (n = 31; mean [SD], 15 [11] mm; range, 0-44 mm).

Figure created using: (1) RStudio Team (2019). RStudio: Integrated Development for R. RStudio, Inc., Boston, MA URL http://www.rstudio.com/, (2) Microsoft PowerPoint, Version 16.52, <https://www.microsoft.com/en-us/microsoft-365/powerpoint>

**Supplementary Table 1. Parameters of Python SimpleITK Registration Toolset**

| Parameter | Value |
| --- | --- |
| Software Version | 1.1.0 |
| Transform Type | BSpline |
| gradientConvergenceTolerance | 1 * 10^-4 |
| numberOfIterations | 10 |
| maximumNumberOfCorrections | 5 |
| maximumNumberOfFunctionEvaluations | 50 |
| costFunctionConvergenceFactor | 1 * 10 ^ 7 |
| Interpolation Type | Linear |
| Convergence metric | Mattes Mutual Information |

**Supplementary Table 2. Geometric Distortion on Portable MRI**

| **Scanner Version^a^** | **Distance from Isocenter (mm)** | **Average Error (mm)^b^** |
| --- | --- | --- |
| Mk 1.2, RC6 | 10 | 0.47 ± 0.09 |
|  | 20 | 0.60 ± 0.10 |
|  | 30 | 0.85 ± 0.12 |
|  | 40 | 1.16 ± 0.17 |
|  | 50 | 1.49 ± 0.21 |
|  | 60 | 1.90 ± 0.25 |
|  | 70 | 2.32 ± 0.28 |
|  | 80 | 2.72 ± 0.33 |
| Mk 1.6, RC8 | 10 | 0.49 ± 0.06 |
|  | 20 | 0.59 ± 0.08 |
|  | 30 | 0.82 ± 0.12 |
|  | 40 | 1.12 ± 0.17 |
|  | 50 | 1.47 ± 0.21 |
|  | 60 | 1.86 ± 0.26 |
|  | 70 | 2.22 ± 0.31 |
|  | 80 | 1.97 ± 0.34 |

^a^ System quality assurance phantoms were scanned by three portable MRI scanners, three runs each. Geometric distortion, centered on the isocenter, was quantified and mapped in accordance to the National Electrical Manufacturers Association (NEMA). The averaged results for each scanner version are summarized above and reported for concentric spheres of 10 mm from the magnet isocenter outwards.

^b^ The mean distance between MLS measurements and the isocenter was 15 mm (**Supplementary Fig. 1d**). The average geometric error for voxels within 20 mm of the isocenter was 0.60 ± 0.10 mm and 0.59 ± 0.08 mm for RC6 and RC8, respectively. These error values are less than the portable MRI voxel size of 1.5 mm, suggesting that geometric distortion had modest impact on portable MRI images and MLS measurements.
